# Supplementary material for: Ambient temperature and genotype differentially affect developmental and phenotypic plasticity in Arabidopsis thaliana
Source: BMC Plant Biol. 2017 Jul 6;17:114. doi: 10.1186/s12870-017-1068-5 (PMC5501000; doi:10.1186/s12870-017-1068-5)
Supplement: Supplementary file 1 — Table of recorded phenotypes and association to phenotype classes. (PDF 12071 kb) [file 12870_2017_1068_MOESM1_ESM.pdf]

Additional file 1: Table of recorded phenotypes and association to phenotype classes
Trait numbers and colors of phenotype classes correspond to Figure 1, Figure 4, and Additional files 3 and 16.

Trait / phentotype class	Morphological marker / time point	Unit	# trait
**Juvenile vegetative stage**			
Germination	radicle emergence	days	1
Seedling establishment	Cotyledons opened fully	days	2
2 rosette leaves	leaf > 1mm in length	days	3
3 rosette leaves	leaf > 1mm in length	days	4
4 rosette leaves	leaf > 1mm in length	days	5
5 rosette leaves	leaf > 1mm in length	days	6
**Juvenile vegetative stage**			
6 rosette leaves	leaf > 1mm in length	days	7
7 rosette leaves	leaf > 1mm in length	days	8
8 rosette leaves	leaf > 1mm in length	days	9
9 rosette leaves	leaf > 1mm in length	days	10
10 rosette leaves	leaf > 1mm in length	days	11
11 rosette leaves	leaf > 1mm in length	days	12
12 rosette leaves	leaf > 1mm in length	days	13
13 rosette leaves	leaf > 1mm in length	days	14
14 rosette leaves	leaf > 1mm in length	days	15
**Reproductive stage**			
Inflorescence emergence	First flower buds visible	days	16
Flowering time_days	Inflorescence > 1cm	days	17
Flowering time_n leaves	Inflorescence > 1cm	no. of leaves	18
Floweri. time_1. flower open	First flower fully opened	days	19
Silique production	Appearance of first silique	days	20
**Morphometric phenotypes**			
Hypocotyl length	7 days old seedlings	pixels	21
Petiole angle	7 days old seedlings	degree	22
Primary root length	7 days old seedlings	pixels	23
Petiole length	20 days old plants	pixels	24
Chlorophyll content (a+ b)	14 days old plants	µg/mg FW	25
Total leaf area (i.e.,. foliar surface)	Inflorescence > 1cm	mm^2	26
Plant height	First silique shattered	cm	27
**Yield-associated phenotypes**			
Seed area	Post harvesting	pixels	28
Seed length	Post harvesting	pixels	29
Seed weight	Post harvesting	µg	30
Total number of seeds per plant	Post harvesting	no./plant	31
Total number of seeds per silique	Post harvesting	no./silique	32
Silique length	Post harvesting	mm	33
Total number of siliques per plant	First silique shattered	no./plant	34
