# Supplementary material for: Ambient temperature and genotype differentially affect developmental and phenotypic plasticity in Arabidopsis thaliana
Source: BMC Plant Biol. 2017 Jul 6;17:114. doi: 10.1186/s12870-017-1068-5 (PMC5501000; doi:10.1186/s12870-017-1068-5)
Supplement: Supplementary file 2 — Sample sizes, identity and geographic origin of analyzed A. thaliana accessions. (PDF 12069 kb) [file 12870_2017_1068_MOESM2_ESM.pdf]

Additional file 2: Sample sizes of phenotypes, Identity, stock numbers and geographic origin of analyzed *A. thaliana* accessions

Stock ID numbers correspond to the European Arabidopsis Stock Centre (Nottingham, UK, www.arabidopsis.info)

accession	Bay-0	C24	Col-0	Cvi-0	Got7	Ler-1	No-0	Rrs-7	Sha	Ws-2
**temperature**	**16**	**16**	**16**	**16**	**16**	**16**	**16**	**16**	**16**	**16**
Germination	20	11	22	20	17	20	11	19	28	20
Seedling establishment	20	17	20	20	20	19	12	20	19	20
2 rosette leaves	18	13	20	17	17	17	12	20	19	15
3 rosette leaves	16	3	19	16	16	18	11	20	19	15
4 rosette leaves	14	3	19	17	17	18	10	20	18	15
5 rosette leaves	13	5	19	15	17	17	9	20	15	14
6 rosette leaves	14	8	19	16	17	17	11	20	19	14
7 rosette leaves	16	15	19	15	17	17	11	20	17	14
8 rosette leaves	16	14	19	15	17	17	11	20	18	14
9 rosette leaves	16	14	19	14	17	17	11	20	18	14
10 rosette leaves	16	14	19	11	17	17	11	20	19	14
11 rosette leaves	15	14	19	10	17	16	10	20	17	14
12 rosette leaves	14	13	19	4	17	14	9	20	16	14
13 rosette leaves	14	11	19	0	17	14	8	20	11	12
14 rosette leaves	11	10	19	0	17	13	7	20	10	12
Inflorescence emergence	10	16	18	14	16	20	14	4	18	19
Flowering time_days	3	12	18	10	16	15	14	0	14	16
Flowering time_leaves	2	13	17	8	0	11	11	0	9	17
Flower.time_1. flower open	6	8	14	10	16	17	7	0	7	12
Silique production	5	5	0	6	0	1	10	0	2	7
Hypocotyl length	17	9	12	9	8	0	7	13	11	17
Petiole angle	9	7	8	6	4	0	4	8	7	7
Length primary root	17	10	14	10	11	0	8	13	11	17
Petiole length	16	16	32	16	16	18	8	10	28	16
Chlorophyll content (a+b)	3	3	0	4	0	3	3	0	4	3
Total leaf area	0	0	0	0	0	0	0	0	0	0
Total number of siliques per plant	9	3	4	7	6	6	7	0	8	8
Plant height	9	3	4	7	6	7	6	10	8	9
Seed area	16	20	17	13	16	20	17	14	21	18
Seed length	20	20	23	20	20	20	20	20	23	20
Seed weight	5	8	9	8	5	8	5	6	9	5
Total number of seeds per plant	5	8	9	8	5	8	5	7	9	5
Total number of seeds per silique	24	19	2	20	10	20	20	10	28	3
Silique length	24	19	2	20	10	20	20	10	28	4

accession	Bay-0	C24	Col-0	Cvi-0	Got7	Ler-1	No-0	Rrs-7	Sha	Ws-2
**temperature**	**20**	**20**	**20**	**20**	**20**	**20**	**20**	**20**	**20**	**20**
Germination	0	0	0	0	0	0	0	0	0	0
Seedling establishment	19	20	20	20	20	20	20	20	20	20
2 rosette leaves	15	18	20	18	20	18	16	19	19	18
3 rosette leaves	14	14	19	18	20	18	16	18	19	18
4 rosette leaves	14	13	19	18	20	18	16	18	19	18
5 rosette leaves	14	12	19	17	20	18	16	18	18	18
6 rosette leaves	14	12	19	17	20	18	16	18	18	18
7 rosette leaves	14	11	19	15	20	18	14	18	17	18
8 rosette leaves	14	11	19	14	20	17	13	18	17	18
9 rosette leaves	14	11	19	14	20	18	7	18	17	17
10 rosette leaves	14	9	19	14	20	18	1	18	17	6
11 rosette leaves	14	9	19	13	20	18	0	18	16	4
12 rosette leaves	13	9	19	12	20	16	0	18	15	1
13 rosette leaves	13	7	19	12	20	18	0	18	15	1
14 rosette leaves	9	7	19	12	20	18	0	18	14	0
Inflorescence emergence	13	11	19	15	6	20	20	17	15	15
Flowering time_days	12	6	19	13	11	18	17	16	17	15
Flowering time_leaves	2	9	17	8	0	5	15	13	9	15
Flower.time_1. flower open	8	4	19	10	7	5	13	14	10	16
Silique production	0	0	0	9	0	9	16	0	11	8
Hypocotyl length	17	13	13	6	13	3	11	17	18	15
Petiole angle	15	11	14	4	12	3	8	12	17	13
Length primary root	19	15	15	8	14	4	11	19	19	17
Petiole length	14	16	16	16	16	12	14	16	16	16
Chlorophyll content (a+b)	0	0	0	0	0	0	0	0	0	0
Total leaf area	6	0	8	5	7	5	7	8	6	8
Total number of siliques per plant	8	2	10	9	0	10	8	9	10	9
Plant height	8	2	10	9	0	10	8	9	10	9
Seed area	22	18	20	18	19	19	20	17	20	17
Seed length	23	20	21	20	20	18	19	20	20	19
Seed weight	8	5	10	7	7	9	7	11	7	10
Total number of seeds per plant	8	5	10	7	7	9	7	11	7	10
Total number of seeds per silique	24	17	9	9	18	20	20	5	20	4
Silique length	24	19	9	9	18	20	20	5	20	4

accession	Bay-0	C24	Col-0	Cvi-0	Got7	Ler-1	No-0	Rrs-7	Sha	Ws-2
**temperature**	**24**	**24**	**24**	**24**	**24**	**24**	**24**	**24**	**24**	**24**
Germination	0	0	0	0	0	0	0	0	0	0
Seedling establishment	20	20	20	20	20	20	12	19	20	20
2 rosette leaves	20	18	20	18	20	20	12	19	20	20
3 rosette leaves	20	18	20	18	20	20	12	19	20	19
4 rosette leaves	20	18	20	18	20	20	11	19	20	19
5 rosette leaves	20	18	20	18	20	20	11	19	20	18
6 rosette leaves	20	18	20	18	20	20	11	19	20	18
7 rosette leaves	20	18	20	18	19	20	9	19	16	17
8 rosette leaves	20	18	20	18	20	20	1	19	9	0
9 rosette leaves	15	17	20	17	20	20	0	19	0	0
10 rosette leaves	12	17	20	17	19	20	0	19	0	0
11 rosette leaves	1	17	20	17	20	8	0	19	0	0
12 rosette leaves	0	17	20	17	20	2	0	19	0	0
13 rosette leaves	0	17	20	17	20	0	0	19	0	0
14 rosette leaves	0	17	20	17	20	0	0	19	0	0
Inflorescence emergence	15	16	20	11	3	20	0	18	7	18
Flowering time_days	7	16	20	12	11	2	0	19	8	18
Flowering time_leaves	16	17	20	12	0	3	12	18	6	19
Flower.time_1. flower open	11	15	20	11	8	20	0	18	2	0
Silique production	0	1	0	1	0	0	0	0	0	0
Hypocotyl length	18	12	14	9	14	6	12	14	12	16
Petiole angle	14	11	13	6	12	4	0	12	12	16
Length primary root	18	12	17	12	14	7	12	14	13	17
Petiole length	24	14	14	16	16	12	0	24	14	16
Chlorophyll content (a+b)	0	0	0	0	0	0	0	0	0	0
Total leaf area	0	0	0	0	0	0	0	0	0	0
Total number of siliques per plant	8	9	8	3	0	10	0	9	6	10
Plant height	8	9	8	4	0	10	0	9	6	10
Seed area	16	17	16	14	11	19	12	17	20	18
Seed length	24	20	20	20	20	20	12	23	20	20
Seed weight	11	7	8	8	10	10	6	8	10	10
Total number of seeds per plant	11	7	8	8	10	10	9	8	10	10
Total number of seeds per silique	24	9	8	9	15	20	12	10	20	4
Silique length	24	9	7	9	15	20	12	10	20	4

accession	Bay-0	C24	Col-0	Cvi-0	Got7	Ler-1	No-0	Rrs-7	Sha	Ws-2
**temperature**	**28**	**28**	**28**	**28**	**28**	**28**	**28**	**28**	**28**	**28**
Germination	24	20	20	20	20	20	19	21	20	20
Seedling establishment	21	19	20	20	20	20	19	19	20	20
2 rosette leaves	21	18	20	19	20	20	19	19	20	20
3 rosette leaves	19	18	20	19	20	20	19	19	18	17
4 rosette leaves	19	18	20	19	20	20	19	19	19	17
5 rosette leaves	19	18	20	19	20	20	18	19	18	17
6 rosette leaves	19	18	19	19	20	20	17	19	18	17
7 rosette leaves	19	18	19	19	20	19	12	19	15	8
8 rosette leaves	17	18	19	19	20	17	1	19	2	0
9 rosette leaves	16	18	18	19	20	15	1	19	0	0
10 rosette leaves	16	18	18	19	20	4	1	19	0	0
11 rosette leaves	1	18	18	19	19	0	1	19	0	0
12 rosette leaves	1	18	17	19	19	0	1	19	0	0
13 rosette leaves	1	18	15	19	19	0	1	18	0	0
14 rosette leaves	1	18	14	19	18	0	1	18	0	0
Inflorescence emergence	14	9	18	0	0	20	11	18	11	11
Flowering time_days	17	9	18	16	9	11	19	18	15	12
Flowering time_leaves	12	8	18	16	0	10	19	11	5	17
Flower.time_1. flower open	12	10	16	0	5	8	19	18	11	20
Silique production	0	0	0	0	0	3	18	0	0	0
Hypocotyl length	16	8	8	8	14	5	16	17	16	15
Petiole angle	15	8	7	9	10	3	1	13	16	15
Length primary root	17	8	12	9	15	7	17	16	17	16
Petiole length	19	15	16	12	12	15	1	21	11	0
Chlorophyll content (a+b)	4	2	0	3	0	3	3	0	3	4
Total leaf area	8	0	20	0	20	14	14	21	11	17
Total number of siliques per plant	16	9	9	0	0	7	9	9	10	8
Plant height	13	9	10	0	0	7	9	9	11	4
Seed area	19	18	18	19	20	20	17	19	19	19
Seed length	25	20	18	20	20	20	19	18	17	20
Seed weight	4	8	10	4	8	10	10	9	9	10
Total number of seeds per plant	4	6	10	4	8	9	10	9	9	10
Total number of seeds per silique	25	20	6	14	10	20	19	11	20	5
Silique length	25	18	6	14	10	20	19	12	20	5

accession	Bay-0	Col-0	C24	Cvi-0	Got-7	Ler-1	No-0	Rrs-7	Sha	Ws-2
Stock ID	N954	N1092	N906	N22682	N22685	N22686	N28564	N22688	N929	N2360
Origin	Germany	USA	Portugal	Cape Verde I:	Germay	Poland	Germany	USA	Tadjikistan	Russia
Latitude	N49	N38	N40	N17	N51	N51	N51	N41	N38	N52
Longitude	E11	W92	W8	W23	E10	E19	E10	W86	E68	E30
