# Supplementary material for: Ambient temperature and genotype differentially affect developmental and phenotypic plasticity in Arabidopsis thaliana
Source: BMC Plant Biol. 2017 Jul 6;17:114. doi: 10.1186/s12870-017-1068-5 (PMC5501000; doi:10.1186/s12870-017-1068-5)
Supplement: Supplementary file 3 — Reaction norm plots of each phenotype for each of the analyzed genotypes. (PDF 12067 kb) [file 12870_2017_1068_MOESM3_ESM.pdf]

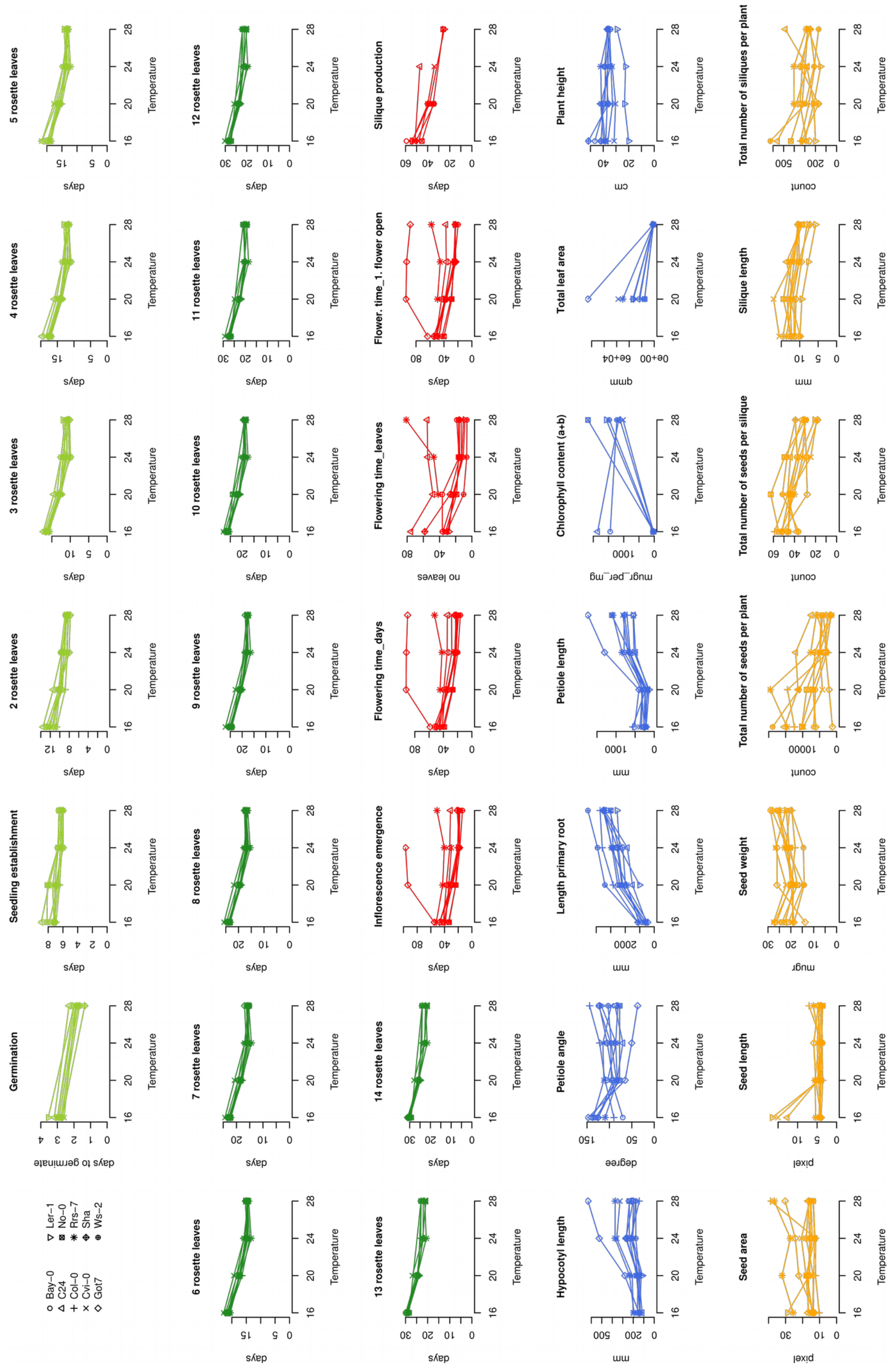

Additional file 3: Reaction norm plots of each phenotype for each of the analyzed genotypes

Data corresponds to the 2-factorial ANOVA of genotype (G), environment (E), and GxE effects presented in Supplementary Table S3.
