# Supplementary material for: Ambient temperature and genotype differentially affect developmental and phenotypic plasticity in Arabidopsis thaliana
Source: BMC Plant Biol. 2017 Jul 6;17:114. doi: 10.1186/s12870-017-1068-5 (PMC5501000; doi:10.1186/s12870-017-1068-5)
Supplement: Supplementary file 10 — Summary of No-0 thermomorphogenesis. (PDF 12069 kb) [file 12870_2017_1068_MOESM10_ESM.pdf]

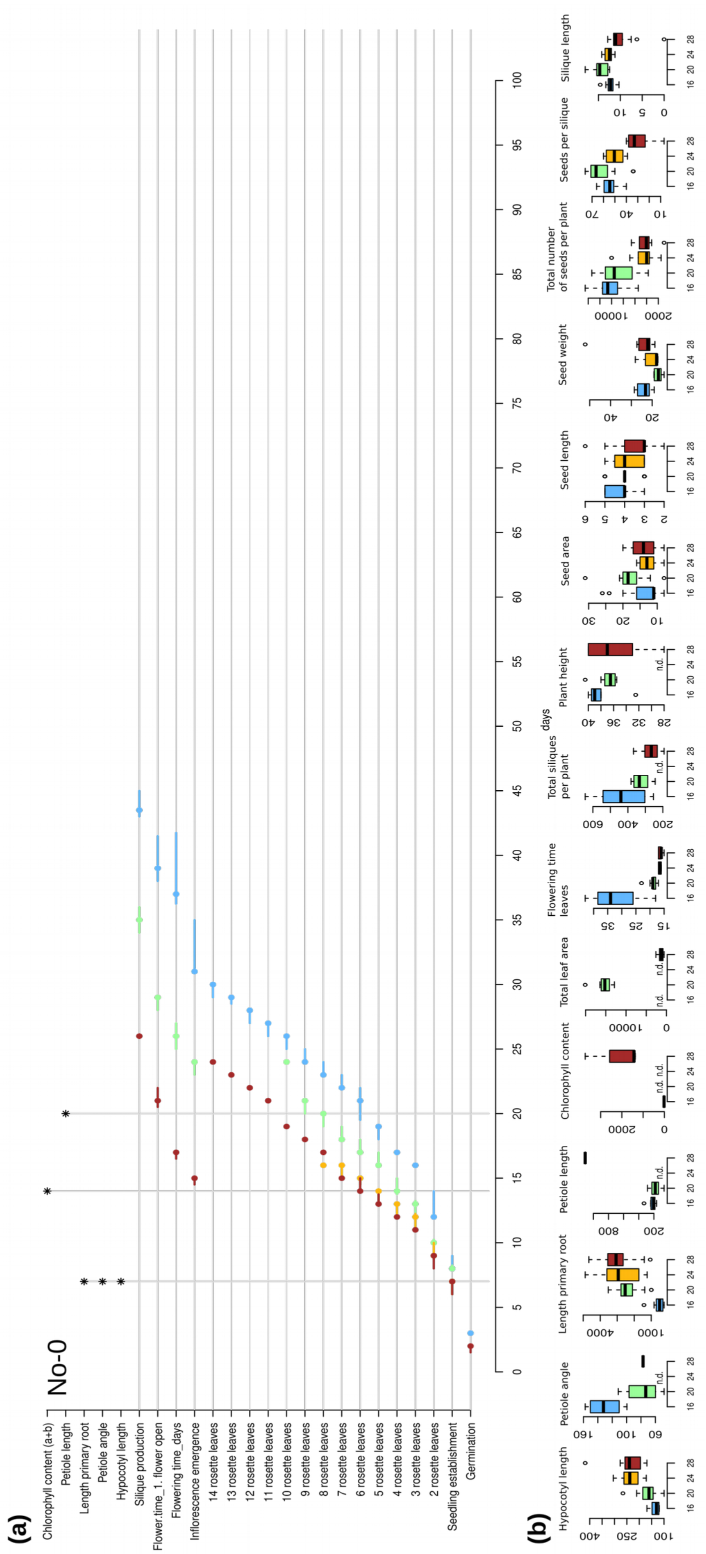

Additional file 10: Summary of No-0 thermomorphogenesis

(a) Developmental timing and (b) quantitative phenotypes of Bay-0 grown at 16 °C (blue), 20 °C (green), 24 °C (yellow), or 28 °C (red). Trait units (x-axis) are noted in Additional file 1. Times of phenotypic assessment for selected traits in (a) are indicated by asterisks.
