# Supplementary material for: Ambient temperature and genotype differentially affect developmental and phenotypic plasticity in Arabidopsis thaliana
Source: BMC Plant Biol. 2017 Jul 6;17:114. doi: 10.1186/s12870-017-1068-5 (PMC5501000; doi:10.1186/s12870-017-1068-5)
Supplement: Supplementary file 14 — Natural variation in developmental timing. (leaves vs. days). (PDF 12069 kb) [file 12870_2017_1068_MOESM14_ESM.pdf]

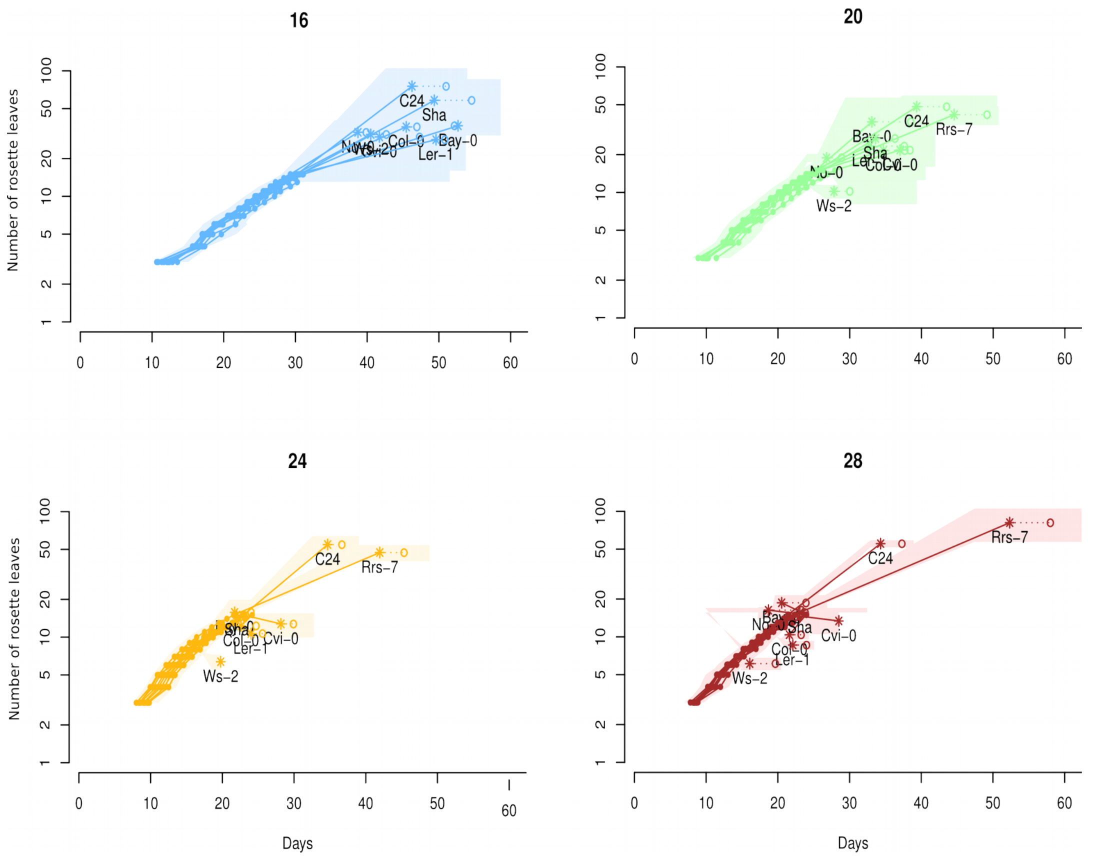

Additional file 14: Natural variation in developmental timing (leaves vs. days)
Plot of the relationship of leaf development over time in developmental timing for each ambient temperature profile. Data points show mean values with filled circles representing vegetative development, asterisks show flowering time_days (bolting) and open circles the time of first flower opening. Shaded areas denote ranges of standard deviations.
