# Supplementary material for: Ambient temperature and genotype differentially affect developmental and phenotypic plasticity in Arabidopsis thaliana
Source: BMC Plant Biol. 2017 Jul 6;17:114. doi: 10.1186/s12870-017-1068-5 (PMC5501000; doi:10.1186/s12870-017-1068-5)
Supplement: Supplementary file 15 — GxE interaction analysis results. (PDF 12069 kb) [file 12870_2017_1068_MOESM15_ESM.pdf]

Additional file 15: GxE interaction analysis

Results of the 2-factorial ANOVA addressing genotype (G), environment (E) and GxE effects. Reaction norms for each phenotype are presented in Additional file 3. ***p < 0.001; **p < 0.01; *p < 0.05

Trait	Source of Variance	Genotype	Environment	GxE	Error
**Germination**	df	9	1	9	372
	Sum SQ	14.92***	116.61***	5.51**	87.52
**Seedling establishment**	df	9	3	27	735
	Sum SQ	84.2***	202.95***	52.44***	313.89
**2 rosette leaves**	df	9	3	27	692
	Sum SQ	104.02***	1265.49***	133.1***	554.09
**3 rosette leaves**	df	9	3	27	662
	Sum SQ	76.28***	3032.94***	137.64***	539.02
**4 rosette leaves**	df	9	3	27	659
	Sum SQ	82.02***	3308.07***	159.52***	607.14
**5 rosette leaves**	df	9	3	27	646
	Sum SQ	121.81***	4155.04***	166.85***	711.84
**6 rosette leaves**	df	9	3	27	655
	Sum SQ	146.94***	5144.48***	136.52***	760.08
**7 rosette leaves**	df	9	3	27	629
	Sum SQ	139.06***	5282.77***	155.61***	619.58
**8 rosette leaves**	df	9	3	25	561
	Sum SQ	442.77***	4693.04***	148.72***	608.96
**9 rosette leaves**	df	9	3	22	534
	Sum SQ	674.91***	4569.79***	143.22***	574.25
**10 rosette leaves**	df	9	3	22	498
	Sum SQ	790.99***	4390.33***	149.63***	616.57
**11 rosette leaves**	df	9	3	20	447
	Sum SQ	763.37***	4038.33***	152.82***	625.28
**12 rosette leaves**	df	9	3	19	420
	Sum SQ	920.74***	3599.42***	151.42***	594.42
**13 rosette leaves**	df	9	3	17	403
	Sum SQ	940.65***	3360.17***	145.15***	588.72
**14 rosette leaves**	df	9	3	16	389
	Sum SQ	1019.36***	3387.69***	154.55***	607.21
**Inflorescence emergence**	df	9	3	24	503
	Sum SQ	57317.87***	20251.24***	21641.29***	6042.78
**Flowering time_days**	df	9	3	25	481
	Sum SQ	112813.21***	16463.8***	29173.91***	7154.8
**Flowering time_leaves**	df	8	3	23	385
	Sum SQ	108107.75***	30695.74***	16859.19***	39414.99
**Flower. time_1. flower open**	df	9	3	23	391
	Sum SQ	82238.45***	16500.55***	22549.53***	5267.36
**Silique production**	df	6	3	6	96
	Sum SQ	4630.95***	4571.85***	283.4***	472.57
**Hypocotyl length**	df	9	3	26	440
	Sum SQ	3238948.9***	1490216.63***	1197320.36***	1289260.71
**Petiole angle**	df	9	3	25	328
	Sum SQ	81097.36***	27122.77***	100398.43***	91660.77
**Length primary root**	df	9	3	26	483
	Sum SQ	85497706.42***	484544690.71***	29350962.77***	224742152.02
**Petiole length**	df	9	3	25	562
	Sum SQ	19364582.03***	34464544.65***	9416311.39***	8703730.03
**Chlorophyll content (a+b)**	df	6	1	6	31
	Sum SQ	6372339.38***	9725371.86***	8697527.37***	5503885.83
**Total leaf area**	df	8	1	7	168
	Sum SQ	19791470218.83***	52198187487.21***	31911951251.09***	2456866415.31
**Plant height**	df	9	3	22	245
	Sum SQ	7663.96***	706.06***	2403.95***	4047.16
**Seed area**	df	9	3	27	670
	Sum SQ	15757.23***	2084.52***	15314.33***	16358.63
**Seed length**	df	9	3	27	762
	Sum SQ	1218.33***	1288.65***	3872.36***	1211.02
**Seed weight**	df	9	3	27	279
	Sum SQ	2106.83***	889.28***	1884.52***	8187.56
**Total number of seeds/plant**	df	9	3	27	280
	Sum SQ	2894602816.9***	2446417943.16***	1629959412.5***	2904762980.9
**Total number of seeds/silique**	df	9	3	27	543
	Sum SQ	13463.79***	41401.43***	14309.34***	42177.64
**Silique length**	df	9	3	27	544
	Sum SQ	1306.39***	1340.46***	390.45***	1502.97
**Total number of siliques/plant**	df	9	3	21	239
	Sum SQ	864294.8***	566244.1***	1276399.97***	1242242.26
