# Supplementary material for: Ambient temperature and genotype differentially affect developmental and phenotypic plasticity in Arabidopsis thaliana
Source: BMC Plant Biol. 2017 Jul 6;17:114. doi: 10.1186/s12870-017-1068-5 (PMC5501000; doi:10.1186/s12870-017-1068-5)
Supplement: Supplementary file 16 — Detailed information on genotype and temperature effects on phenotypic variation. (PDF 12069 kb) [file 12870_2017_1068_MOESM16_ESM.pdf]

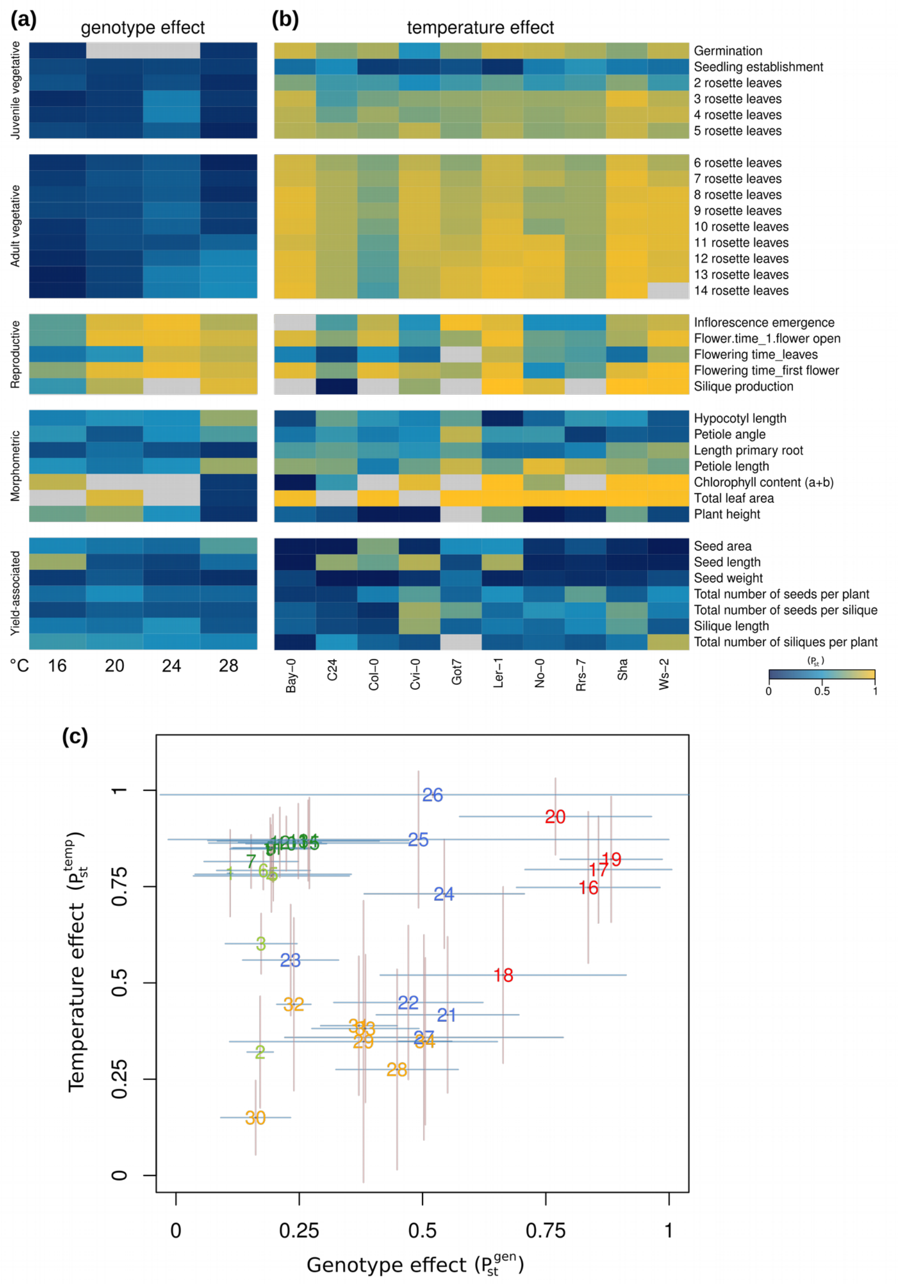

Additional file 16: Detailed information on genotype and temperature effects on phenotypic variation

(a) P_{st}^{gen} for individual temperatures were calculated across all ten genotypes and (b) P_{st}^{temp} values were calculated for all accessions across all four temperatures to assess genotype and temperature effects on phentotypic variation, respectively. Missing data is shown in light gray. (c) Scatter plot of mean P_{st}^{gen} and mean P_{st}^{temp} values including standard deviation, corresponding to data presented in Fig. 4b.
