# Supplementary material for: Ambient temperature and genotype differentially affect developmental and phenotypic plasticity in Arabidopsis thaliana
Source: BMC Plant Biol. 2017 Jul 6;17:114. doi: 10.1186/s12870-017-1068-5 (PMC5501000; doi:10.1186/s12870-017-1068-5)
Supplement: Supplementary file 17 — Temperature effect on yield. (PDF 12069 kb) [file 12870_2017_1068_MOESM17_ESM.pdf]

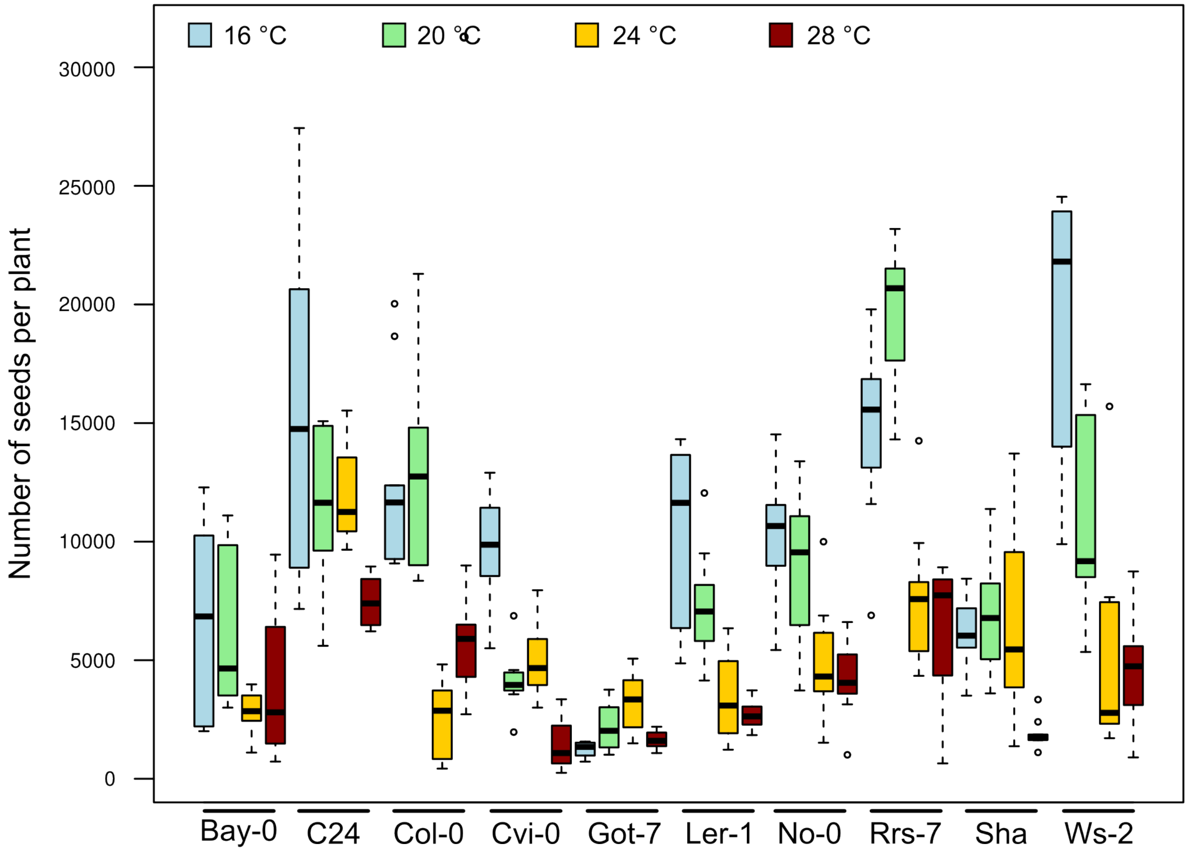

Additional file 17: Temperature effect on yield (absolute values)
Total number of seeds per plant. Box plots show median and interquartile ranges (IQR), outliers (> 1.5 times IQR) are shown as circles.
