# Supplementary material for: Ambient temperature and genotype differentially affect developmental and phenotypic plasticity in Arabidopsis thaliana
Source: BMC Plant Biol. 2017 Jul 6;17:114. doi: 10.1186/s12870-017-1068-5 (PMC5501000; doi:10.1186/s12870-017-1068-5)
Supplement: Supplementary file 18 — Correlations among temperature responses in individual accessions. (PDF 12069 kb) [file 12870_2017_1068_MOESM18_ESM.pdf]

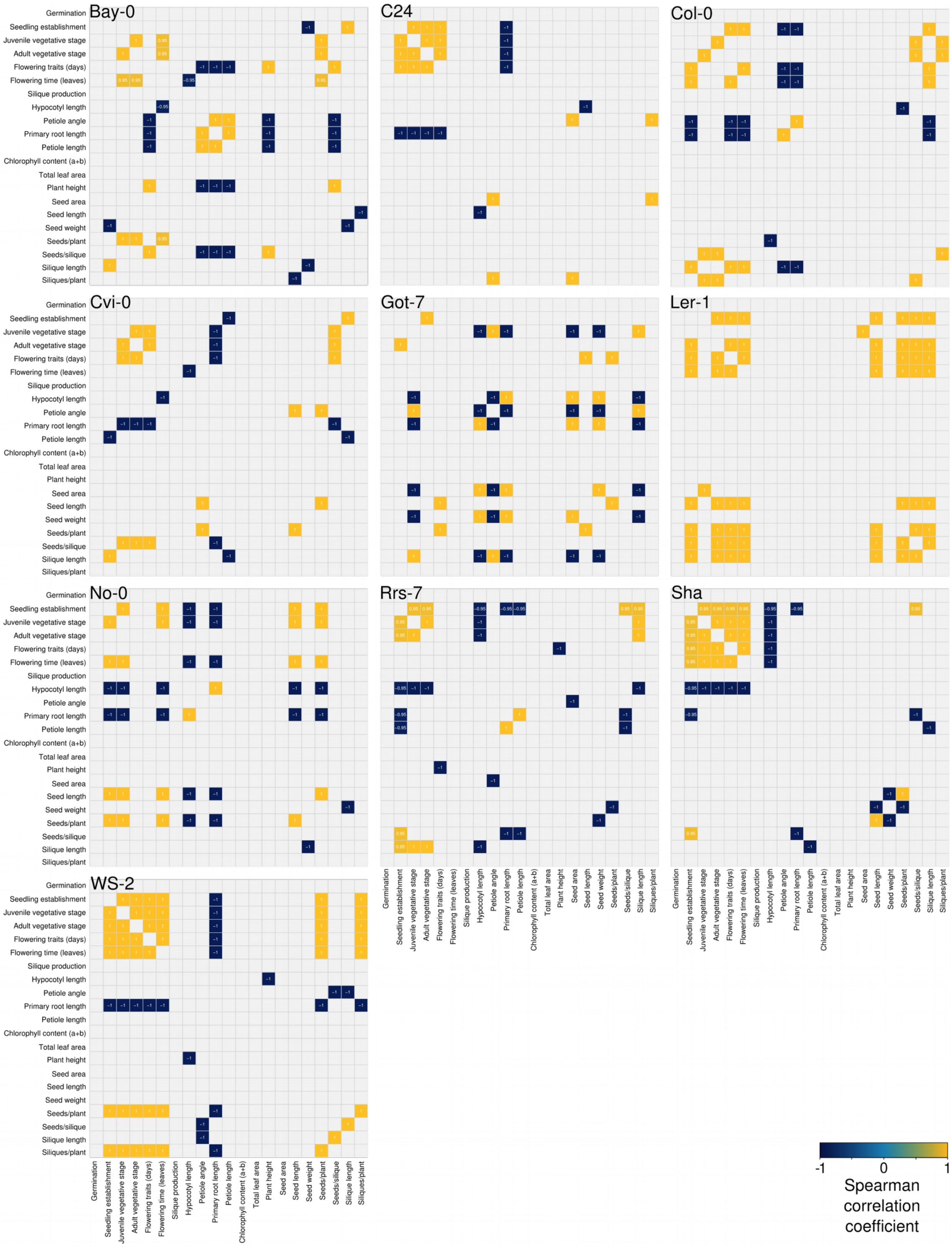

Additional file 18: Correlations among phenotypic temperature responses
Heatmap of Spearman correlation values of trait value comparisons among all phenotype pairs. Empty squares correspond to correlation coefficients if they did not pass testing for significance and correction for multiple testing (P < 0.1). Data corresponds to example data presented in Fig. 5c.
